# External Validation of the Bilirubin–Atazanavir Nomogram for Assessment of Atazanavir Plasma Exposure in HIV-1-Infected Patients

**DOI:** 10.1208/s12248-012-9440-8

**Published:** 2012-12-07

**Authors:** Dinko Rekić, Daniel Röshammar, Martin Bergstrand, Joel Tarning, Andrea Calcagno, Antonio D’Avolio, Vidar Ormaasen, Marie Vigan, Aurélie Barrail-Tran, Michael Ashton, Magnus Gisslén, Angela Äbelö

**Affiliations:** 1 Unit for Pharmacokinetics and Drug Metabolism, Department of Pharmacology, Sahlgrenska Academy, University of Gothenburg, Gothenburg, Sweden; 2 AstraZeneca R&D Mölndal, Mölndal, Sweden; 3 Department of Pharmaceutical Biosciences, Uppsala University, Uppsala, Sweden; 4 Mahidol–Oxford Tropical Medicine Research Unit, Faculty of Tropical Medicine, Mahidol University, Bangkok, Thailand; 5 Centre for Tropical Medicine, Nuffield Department of Clinical Medicine, University of Oxford, Oxford, UK; 6 Department of Infectious Diseases, University of Torino, Torino, Italy; 7 Department of Infectious Diseases, Clinical Pharmacology and Pharmacogenetics Laboratory, University of Torino, Torino, Italy; 8 Department of Infectious Diseases, Division of Internal Medicine, Oslo University Hospital, Oslo, Norway; 9 Université Paris Diderot, Sorbonne Paris Cité, UMR 738, 75018 Paris, France; 10 INSERM, UMR 738, 75018 Paris, France; 11 Department of Clinical Pharmacy, Assistance Publique Hôpitaux de Paris, Hôpitaux Universitaires Paris-Sud, Le Kremlin Bicêtre, France; 12 Faculté de Pharmacie, Université Paris Sud, Chatenay Malabry, France; 13 Department of Infectious Diseases, Sahlgrenska University Hospital, University of Gothenburg, Gothenburg, Sweden; 14 Po Box 431, 405 30 Gothenburg, Sweden

**Keywords:** atazanavir, bilirubin, nomogram

## Abstract

Atazanavir increases plasma bilirubin levels in a concentration-dependent manner. Due to less costly and readily available assays, bilirubin has been proposed as a marker of atazanavir exposure. In this work, a previously developed nomogram for detection of suboptimal atazanavir exposure is validated against external patient populations. The bilirubin nomogram was validated against 311 matching bilirubin and atazanavir samples from 166 HIV-1-infected Norwegian, French, and Italian patients on a ritonavir-boosted regimen. In addition, the nomogram was evaluated in 56 Italian patients on an unboosted regimen. The predictive properties of the nomogram were validated against observed atazanavir plasma concentrations. The use of the nomogram to detect non-adherence was also investigated by simulation. The bilirubin nomogram predicted suboptimal exposure in the patient populations on a ritonavir-boosted regimen with a negative predictive value of 97% (95% CI 95–100). The bilirubin nomogram and monitoring of atazanavir concentrations had similar predictive properties for detecting non-adherence based on simulations. Although both methods performed adequately during a period of non-adherence, they had lower predictive power to detect past non-adherence episodes. Using the bilirubin nomogram for detection of suboptimal atazanavir exposure in patients on a ritonavir-boosted regimen is a rapid and cost-effective alternative to routine measurements of the actual atazanavir exposure in plasma. Its application may be useful in clinical settings if atazanavir concentrations are not available.

## INTRODUCTION

Like other protease inhibitors (PI), atazanavir displays large interindividual pharmacokinetic variability resulting in variable drug exposure between patients. Trough concentrations above the minimal effective concentration (MEC) of 0.2 μmol/L (150 ng/ml) are recommended as a suitable target for atazanavir plasma monitoring (1). Monitoring of atazanavir drug concentrations has mainly been recommended in special cases when there is a substantial risk of drug–drug and/or food–drug interactions (1). However, there is evidence that routinely applied atazanavir plasma concentration monitoring in combination with applied pharmacokinetic analysis could improve atazanavir-based therapy (2), while other studies have failed to demonstrate any benefit (3).

The use of bilirubin as biological marker of atazanavir adherence, exposure, or treatment outcome has previously been investigated (3–7). The reason for this is that atazanavir inhibits intrahepatocellular bilirubin glucuronidation by inhibition of glucuronosyltransferase 1A1 (UGT1A1), resulting in increased bilirubin levels (8). Karlström *et al.* reported that average bilirubin increase from baseline, in patients on atazanavir monotherapy, was significantly lower in subjects with virological failure compared to those without whereas there were no differences in atazanavir *C*_trough_ between the two groups (7). Similarly, Petersen *et al.* demonstrated significantly higher bilirubin increments in patients with successful viral suppression than in patients failing atazanavir-based combination therapy (5). Bilirubin is routinely measured in clinical settings and at a low cost compared to atazanavir plasma measurement. The suggested approach of using bilirubin as a biomarker for atazanavir exposure would therefore be of advantage when cost, speed, or availability of assays is an issue (4, 5).

The quantitative relationship between atazanavir exposure and bilirubin was recently described using a population pharmacokinetic–pharmacodynamic (PKPD) model (4). Based on the model, a nomogram was developed and designed to predict suboptimal atazanavir exposure (4). Primarily, the aim of this work was to validate the bilirubin nomogram in external patient populations on a ritonavir-boosted regimen. A secondary aim was to investigate the predictive properties of the nomogram compared to traditional plasma monitoring, for diagnosing treatment adherence in a simulated population of adherent and non-adherent patients. Additionally, the predictive properties were investigated in a population of patients treated with an unboosted atazanavir regimen.

## METHODS

### Study Populations

The Italian patients were part of the therapeutic drug monitoring programs at University of Torino. Both ritonavir-boosted (*n* = 56) and unboosted (*n* = 56) patients were included, but the data were analyzed separately. All patients on an atazanavir/ritonavir regimen were on a 300/100-mg QD regimen except two who were on a 200- and 400-mg QD-based regimen, respectively. The regimens of the unboosted patients varied from 200 mg BID to 400 mg QD. The backbone therapies varied according to clinical practice. The bilirubin steady-state samples were collected on average at 09:23 (±1:05), while baseline samples were collected between 8:00 and 11:00 am.

The Norwegian patients (*n* = 76) were part of the HIV monitoring program at Oslo University Hospital. The data were extracted from the Thematic Biobank “Infectious Diseases.” All patients were administered an atazanavir/ritonavir (300/100 mg QD)-containing regimen. The backbone therapy varied according to clinical practice. The average times for bilirubin baseline and steady-state sampling were 10:13 am (±1:38) and 09:44 (±1:22) am, respectively.

The French patients were part of the ANRS 134-COPHAR 3 study (9). Of the 35 patients recruited to the study, one was excluded from this analysis due to a missing bilirubin baseline measurement. The patients were administered 300 mg atazanavir QD, 100 mg ritonavir QD, and tenofovir/emtricitabine (245/200 mg) for 24 weeks. Matching bilirubin and atazanavir observations were available at week 4, 8, 16, and 24. On average, the samples were collected 18.27 h after dose.

Full demographics of the populations are shown in Table I.

**Table I:** Patient Demographics and Clinical Characteristics

Characteristic	Italian				French		Norwegian	
	Unboosted ATZ		Boosted ATZ		Boosted ATZ		Boosted ATZ	
	Value	Mean (±SD)	Value	Mean (±SD)	Value	Mean (±SD)	Value	Mean (±SD)
Number of patients	56	–	56	–	34		76	–
Male patients	38	–	33	–	28		59	–
Female patients	18	–	23	–	6		17	–
Number of atazanavir concentrations at steady state	103	–	84	–	130		97	–
Number of bilirubin samples at baseline	56	–	56	–	34		76	–
Number of bilirubin samples at steady state	103	–	84	–	130		97	–
Body weight (kg)	–	71 (15.3)	–	71 (14)	–	72.0 (9.9)	–	71.9 (13.9)
Age (years)	–	47 (13)	–	43 (11)	–	37 (9)	–	41 (10)
Bilirubin at baseline (μmol/L)	–	10.0 (7.5)	–	9.5 (3.8)	–	9.4 (3.5)	–	7.5 (3.7)
Bilirubin at steady state (μmol/L)	–	24.5 (17.8)	–	36.6 (23.8)	–	42.5 (26.2)	–	32.2 (19.1)
Atazanavir plasma concentrations below MEC	45	–	4	–	9	–	4	–
CD4 cell count at baseline, (× 10^6^/μL)	–	401 (199)	–	372 (224)	–	290 (76)	–	268 (168)
Detectable viral load at baseline	35	–	53	–	34	–	61	–

*ATZ* atazanavir, *SD* standard deviation

### Application of the Nomogram

The bilirubin baseline and steady-state levels were analyzed with the bilirubin–atazanavir nomogram. Individual baseline bilirubin concentrations were plotted on the *x*-axis while the individual bilirubin steady-state concentrations were plotted on the *y*-axis. Observations on the solid black area of the nomogram were identified as corresponding to atazanavir exposure below the minimum effective concentration (MEC). Observations correctly and incorrectly identified as below MEC were considered to be true positives (TP) and false positives (FP), respectively. Observations correctly and incorrectly identified as above MEC were considered to be true negatives (TN) and false negatives (FN), respectively.

The proposed nomogram is in many ways equivalent to a diagnostic test for a disease, *e.g.*, a HIV test. A positive test result can indicate presence of some disease, in contrast to a negative test result which indicates absence of the disease in question. In this case, a positive nomogram result indicates presence of suboptimal exposure or non-adherence. A number of statistical metrics, commonly used to evaluate the performance of diagnostic tests, can be calculated based on the TP, FP, TN, and FN values, *e.g.*, specificity, sensitivity, accuracy positive predictive value (PPV), and negative predictive value (NPV) (Table II) (10, 11). The term NPV is the proportion of patients with negative test results that are correctly diagnosed while the term PPV is the proportion of patients with positive test results that are correctly diagnosed (10, 11).

**Table II:** Equations and Interpretations of the Metrics Used to Describe the Predictive Properties of the Nomogram

Statistical metric	Interpretation
Negative result (*N*)	The nomogram identifies the sample to be above MEC of atazanavir.
True N (TN)	The sample is correctly predicted to be above MEC.
False N (FN)	The sample is incorrectly predicted to be above MEC.
Positive result (*P*)	The nomogram identifies the sample to be below MEC of atazanavir.
True P (TP)	The sample is correctly predicted to be below MEC.
False P (FP)	The sample is incorrectly predicted to be below MEC.
$\mathrm{Specificity}=\frac{\mathrm{TN}}{{\mathrm{TP}+\mathrm{FP}}}$	Specificity of the nomogram is the probability of a true negative result when the atazanavir sample is over MEC.
$\mathrm{Sensitivity}=\frac{\mathrm{TP}}{{\mathrm{TP}+\mathrm{FN}}}$	Sensitivity of the nomogram is the probability of a true positive result when the atazanavir sample is under MEC
$\mathrm{Accuracy}=\frac{{\mathrm{TP}+\mathrm{TN}}}{{\mathrm{TP}+\mathrm{TN}+\mathrm{FP}+\mathrm{FN}}}$	Accuracy is the proportion of all correctly predicted observations for the nomogram.
$\mathrm{Negative}\;\mathrm{predictive}\;\mathrm{value}=\frac{\mathrm{TN}}{{\mathrm{TN} + \mathrm{FN}}}$	NPV is the probability of a negative test to be true negative.
$\mathrm{Positive}\;\mathrm{predictive}\;\mathrm{value}=\frac{\mathrm{TP}}{{\mathrm{TP} + \mathrm{FP}}}$	PPV is the probability of a positive test to be true positive.

*MEC* minimum effective concentration (0.2 μmol/L)

### Simulation-Based Exploration of the Predictive Properties of the Nomogram

**Fig. 1:**
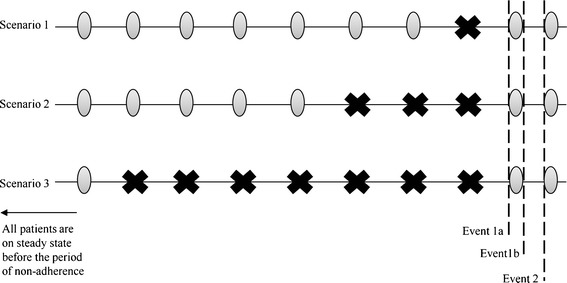
Study design for the simulation-based validation. The *crosses* represent days of non-adherence to atazanavir while the *ellipsoids* represent administered doses. The *dashed lines* represent sampling/monitoring events. *Event 1a*, patients are monitored/sampled 24 h after a period of non-adherence. *Event 1b*, patients are monitored/sampled 1 h after an atazanavir dose event following a period of non-adherence. *Event 2*, patients are monitored/sampled 48 h after a period of non-adherence

In order to evaluate if the nomogram can predict non-adherence, it was applied to data from 1,000 simulated patients of whom 10% were non-adherent to therapy. Simulations of three scenarios were performed (Fig. 1) based on the previously developed PKPD model (4).

Scenario 1 describes a patient forgetting or actively deciding not to take one single atazanavir dose after a period of full adherence to therapy. Patients not adherent to treatment during three consecutive days subsequent to a period of full adherence are portrayed in scenario 2. In scenario 3, it was assumed that the patient is not adhering to therapy for an extended period of time with seven consecutively missed doses following a period of full adherence. In all three scenarios, it was assumed that the patient was scheduled for an atazanavir plasma monitoring event before the scheduled dosing time at the day following a past period of non-adherence (event 1a), or on the second day after a non-adherence period (event 2). After the period of non-adherence, the patients are completely adherent to therapy. To account for situations where patients adhere to therapy in connection to clinic visits, a separate event was simulated for each scenario where the patient takes an atazanavir dose 1 h before the scheduled clinic visit without informing the physician (event 1b). In all scenarios, bilirubin at baseline and atazanavir plasma concentrations with matching bilirubin concentrations at steady state were simulated for all patients at the described monitoring events.

Patients were considered to be non-adherent when the nomogram identified a simulated bilirubin sample below the cutoff corresponding to an atazanavir plasma concentration below the MEC. Similarly, patients were considered non-adherent when simulated atazanavir plasma concentrations were below MEC. Correctly identified non-adherent and adherent patients were labeled as TP and TN, respectively. Incorrectly identified non-adherent and adherent patients were labeled FP and FN, respectively. The ability to detect non-adherence from bilirubin levels of the nomogram and atazanavir plasma measurements were compared. All simulations were performed using NONMEM 7 (ICON Development Solutions, Ellicot City, MD, USA) with PsN version 3.4.2 (12, 13).

### Computation of Predictive Properties

The epiR package in R (2.14) was used for calculation of specificity, sensitivity, accuracy, PPV, and NPV for the external and simulation-based validation (Table II). The Norwegian, French, and Italian ritonavir-boosted patients were analyzed both separately and together. The unboosted Italian patients were analyzed separately

## RESULTS

### External Validation of the Bilirubin–Atazanavir Nomogram

**Fig. 2:**
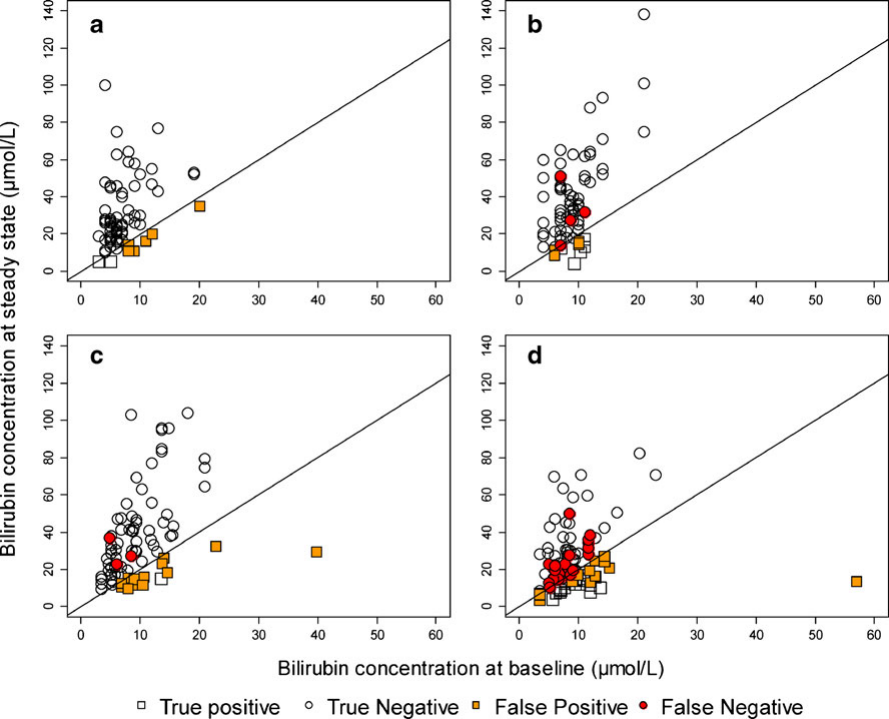
The bilirubin nomogram applied on Norwegian (**a**), French (**b**), ritonavir-boosted Italian (**c**), and unboosted Italian (**d**) patients. Observations below the *full line* are predicted to have atazanavir concentrations below the MEC of 0.2 μmol/L (150 ng/ml). The *white points* denote correct predictions. The *red* and the *orange points* denote incorrect predictions

**Fig. 3:**
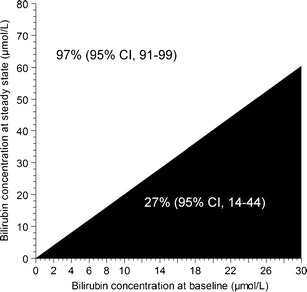
External validation results of the nomogram. The *black area* represents bilirubin steady-state levels associated with atazanavir exposure below the MEC of 0.2 μmol/L. The percentages and the confidence intervals (95% CI) in the *white* and the *black area* represent the probability of the nomogram to be correct when predicting an observation to be above or below MEC, respectively

Out of 311 atazanavir observations in 166 patients on a ritonavir-boosted atazanavir regimen, 294 were above MEC. The nomogram identified 267 of these bilirubin observations correctly (TN) while 27 observations were incorrectly identified as below MEC (FP). Seven observations were incorrectly identified as above MEC (FN) while ten observations were correctly identified as below MEC (TP). Metrics for the unboosted and the ritonavir boosted subpopulations are shown in Table III. In general, the nomogram predictions were significantly worse for unboosted patients (NPV, 70% [95% CI 0.57–0.82]). The bilirubin observations for the various populations are plotted on the nomogram in Fig. 2. The NPV and the PPV from the combined analysis of all ritonavir-boosted patients are shown on the white and the black areas of the nomogram, respectively (Fig. 3).

**Table III:** Summary of the Bilirubin Nomogram's Predictive Properties in Various HIV-1 Patient Populations

Parameter	Italian				French		Norwegian		Total	
	Unboosted ATZ		Boosted ATZ		Boosted ATZ		Boosted ATZ		Boosted ATZ	
	Value	95% CI	Value	95% CI	Value	95% CI	Value	95% CI	Value	95% CI
Specificity	0.79	(0.66–0.89)	0.81	(0.71–0.89)	0.95	(0.91–0.99)	0.92	(0.85–0.97)	0.91	(0.87–0.94)
Sensitivity	0.56	(0.40–0.78)	0.25	(0.01–0.80)	0.55	(0.21–0.86)	1.00	(0.28–1.00)	0.59	(0.33–0.82)
Accuracy	0.69	(0.59–0.78)	0.79	(0.68–0.87)	0.93	(0.87–0.97)	0.93	(0.86–0.97)	0.89	(0.85–0.92)
PPV	0.68	(0.50–0.82)	0.06	(0.002–0.3)	0.50	(0.19–0.81)	0.36	(0.11–0.69)	0.27	(0.14–0.44)
NPV	0.70	(0.57–0.80)	0.96	(0.88–0.99)	0.97	(0.92–0.99)	1.00	(0.94–1.00)	0.97	(0.95–0.99)

*PPV* positive predictive value, *NPV* negative predictive value, *CI* confidence interval, *ATZ* atazanavir

### Simulation-Based Exploration of the Predictive Properties of the Nomogram

**Fig. 4:**
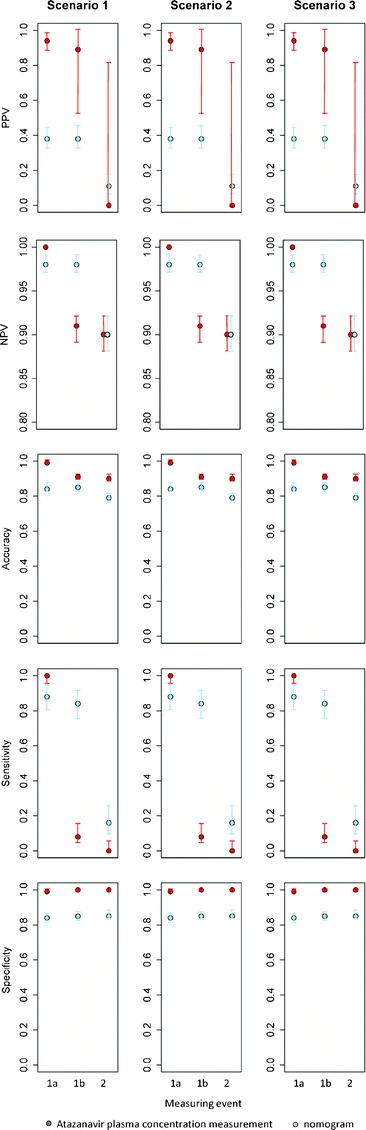
Summary of predictive properties of the bilirubin nomogram (*circles*) and atazanavir drug monitoring (*triangles*) based on simulations of 1,000 virtual patients. The *circles* represent the median while the bars represent the 95% confidence interval. Colors *red* and *blue* represent the atazanavir concentration measurement and bilirubin, respectively. *PPV* positive predictive value, *NPV* negative predictive value. The scenarios and the events are explained in the “Methods” section

The predictive properties of the bilirubin nomogram and when using measured atazanavir concentrations in assessing treatment adherence are shown in Fig. 4. There was some but no consistent difference in performance for the two methods. In terms of NPV, both methods performed adequately at event 1a in all cases (>98%). The bilirubin nomogram had significantly higher NPV at event 1b compared to atazanavir plasma measurement. At event 2, 48 h after the non-adherent period, both methods had a NVP of 90% which is in line with the simulated prevalence (10%) of non-adherent patients.

PPV at events 1a and 1b was higher for atazanavir plasma measurement compared to the nomogram. However, in each scenario, the atazanavir plasma measurement was less reliable at events 1b and 2, identifying only zero and eight out of a total of 100 non-adherent patients, respectively. This is reflected by the wide confidence intervals for PPV in Fig. 4.

Sensitivity at event 1a was higher for atazanavir plasma measurement compared to the nomogram, 88% (95% CI 80–94) *vs.* 100% (95 CI 95–100). At event 1b, the nomogram outperformed the atazanavir plasma measurement in all scenarios, 84% (95% CI 75–90) *vs.* 8% (95% CI 4–15). At event 2, both methods performed poorly in terms of sensitivity (<16%).

Accuracy decreased slightly going from event 1a to 1b and 2 in all scenarios for the atazanavir monitoring method. The nomogram performed more consistently at different events albeit with slightly lower accuracy. Using the atazanavir plasma measurement, specificity was consistently very high (>99%) at all events and scenarios while the nomogram performance was somewhat lower.

## DISCUSSION

Although not routinely recommended, plasma monitoring of atazanavir has been suggested as a tool for improving antiretroviral efficacy and safety (1, 2). The cost of plasma concentration quantification can potentially have a role in the underuse of drug monitoring. The bilirubin nomogram may therefore be of benefit when situations call for atazanavir monitoring in particular when there is substantial risk of drug–drug interactions influencing atazanavir exposure or adherence follow-up.

The bilirubin nomogram was developed based on a previously developed PKPD model describing atazanavir concentration-dependent elevation in bilirubin levels (4). The nomogram is intended for use in routine care of HIV-1-infected patients on an atazanavir/ritonavir-based treatment. The *y*- and *x*-axes of the nomogram represent bilirubin levels at steady state (after atazanavir treatment initiation) and at baseline (before treatment initiation), respectively. In brief, a bilirubin baseline sample should be collected between 9 am and 3 pm before initiation of therapy since bilirubin levels are stable between those hours (14). After 3 pm, the bilirubin levels drop substantially due to circadian variation (4). The baseline bilirubin concentration is plotted on the *x*-axis of the nomogram while the new steady-state concentration sampled at least 2 weeks after treatment initiation is plotted on the *y*-axis. If a value is located within the lower (black solid) area of the nomogram, then the sample is expected to correspond to a drug concentration below the MEC value for atazanavir exposure. In all other areas of the nomogram, the sample is considered to correspond to a drug concentration above the MEC value. Factors known to influence bilirubin can have a confounding effect on the nomogram predictions. The most influential confounder would be one causing elevated bilirubin levels and thereby increasing the risk of false negatives, *i.e.*, failure to identify a patient below the MEC. Gilbert's syndrome is such a confounder, where the UGT1A1 gene allele *28 has been shown to increase the risk of hyperbilirubinemia with some PI-based treatments (15, 16). An undiagnosed and not clinically manifested Gilbert's syndrome could thus potentially confound the results of the nomogram. The use of the nomogram on patients with high levels of baseline bilirubin levels is therefore not advised. One Italian patient, identified as FP, had a very high baseline bilirubin concentration (40 μmol/L). After initiation of atazanavir therapy, the bilirubin concentrations decreased to 30 μmol/L.

Due to a high number of false positives, the PPV was significantly lower in Italian patients than in French/Norwegian patients. The influence of HIV/hepatitis C coinfection on the nomogram performance has not been investigated. Hence, differences in HIV/hepatitis C coinfection prevalence between the Italian and the French/Norwegian have not been investigated nor accounted for. The Italian patients had a HIV/hepatitis C coinfection prevalence of 23.3. None of the French patients in this analysis were infected with hepatitis B or C due to the exclusion criteria of the ANRS 134-COPHAR 3 study. Although the prevalence in the Norwegian patients was not known, a similar cohort from Norway had a prevalence of 6% (17).

The UGT1A1*28 allele, associated with Gilbert's syndrome, has a prevalence of 16% in Europeans, 12% in Indians, 8% in Egyptians, 28% in African–Americans while Chinese and Japanese have low frequencies of the allele (18). Neither race nor ethnicity was included in this analysis, and it is assumed that all three cohorts include patients with various racial and ethnical backgrounds. Hence, it can also be assumed that a large number of patients are carriers of the UGT1A1*28 allele. Other possible confounders, such as other drugs or conditions interfering with bilirubin metabolism, were not investigated in this study. Starvation, stress, physical activity, severe blood loss, obstructive bile duct, sepsis, trauma, congestive heart failure, and hemolysis are known factors to influence bilirubin levels in humans (18). However, the patients used for the external validation of the nomogram represent typical HIV-1 patient populations. The nomogram is therefore expected to perform similarly in other typical HIV-1-infected populations.

The credibility of a negative prediction by a test, such as the nomogram, is reflected by the NPV. Applied to three external populations, the NPV for the nomogram was estimated at 97% (95% CI 91–99). It should be noted that the NPV can be affected by the prevalence of suboptimal exposure and vary amongst populations with different prevalence. Here, the prevalence of suboptimal atazanavir exposure was similar for the Norwegian, Italian, and French populations: 4%, 5%, and 7%, respectively. The reason for the small discrepancy between French and Italian/Norwegian patients could be attributed to the study design. The French patients were included in a clinical trial and observed at four occasions within 6 months of follow-up after treatment initiation. The Italian and Norwegian patients were part of a monitoring program and had only a few observations per individual. Consequently, a consistently non-adherent patient would influence the prevalence estimate more in the French than in the Norwegian/Italian population.

The nomogram was designed to be used as a first screening tool for suboptimal exposure and non-adherence. Given the nature of drug monitoring as a screening tool for suboptimal exposure and non-adherence, it is arguable that the confidence in a negative result (exposure over MEC) is of greater importance than the confidence of a positive result (exposure below MEC). A sample identified by the nomogram as positive can be reanalyzed, and the atazanavir concentration can be quantified, giving a definitive answer if the low bilirubin sample was a true- or a false-positive result in comparison to atazanavir concentration.

The nomogram was developed using data from ritonavir-boosted patients. As evident by the current data, the nomogram performs poorly when used on patients on an unboosted atazanavir regimen. Ritonavir-boosted patients are known to have higher bilirubin levels than unboosted which also was observed here (19). Ritonavir may also affect the access of atazanavir to the intracellular space of hepatocytes though inhibition of the OATP1B1 transporter (20). For this reason, there may be a need for a new nomogram, developed based on data from patients on an unboosted atazanavir regimen.

Since the reason for low atazanavir exposure in the external dataset was unknown, a simulation study based on the developed PKPD model was designed where 10% of the virtual patients were non-adherent to therapy. Three different clinically plausible scenarios of non-adherence were evaluated under three different sampling schedules. The predictive properties of the nomogram were compared to the simulated atazanavir plasma concentrations. In general, there were no differences between the scenarios; furthermore, the nomogram and the plasma concentration measurements were similar in their ability to predict non-adherence in patients in the ideal case when the patient is sampled directly after the period of non-adherence (event 1a). If a patient decides to take an atazanavir dose before the sampling event without informing the physician, the atazanavir plasma measurement would not indicate non-adherence whereas the nomogram would (event 1b). Neither of the two approaches was successful in detecting non-adherence when the sampling event took place 48 h after a period of non-adherence (event 2). These results were expected since bilirubin half-life was previously estimated at 8.2 h when atazanavir is at steady state (4).

Based on the simulations, it is questionable if plasma concentration monitoring is of use to assess non-adherence other than in the ideal case (event 1a). For the ideal case, the NPV was estimated above 98% for both the methods resulting in good predictive properties. The use of atazanavir plasma monitoring as a tool to motivate adherence in patients has not been addressed here. The simulation study was solely designed to verify if non-adherence could be detected. However, other sources of unexpected low plasma exposure such as food–drug and/or drug–drug interactions could also be explored in a similar manner.

In conclusion, this work demonstrates that the bilirubin nomogram is a rapid, cost-effective, and useful alternative to atazanavir plasma concentration monitoring for the use as a screening tool for suboptimal atazanavir exposure and non-adherence in patients. The bilirubin nomogram may diminish some obstacles in implementing drug monitoring of atazanavir-based antiretroviral therapy, such as lack of laboratories and the high cost of atazanavir measurement. The simplistic design of the nomogram and the straightforward interpretation of the results should facilitate its implementation in clinical practice.
